# Pristine GaFeO_3_ Photoanodes with Surface Charge Transfer Efficiency of Almost Unity at 1.23 V for Photoelectrochemical Water Splitting

**DOI:** 10.1002/advs.202205907

**Published:** 2023-01-19

**Authors:** Xin Sun, Min Wang, Hai‐Fang Li, Linxing Meng, Xiao‐Jun Lv, Liang Li, Meicheng Li

**Affiliations:** ^1^ State Key Laboratory of Alternate Electrical Power System with Renewable Energy Sources, School of New Energy North China Electric Power University Beijing 102206 China; ^2^ School of Physical Science and Technology Jiangsu Key Laboratory of Thin Films Center for Energy Conversion Materials & Physics (CECMP) Soochow University Suzhou 215006 China

**Keywords:** defect‐inactive photoanodes, GaFeO_3_ thin films, oxygen vacancy, surface charge transfer, surface recombination

## Abstract

Oxide‐based photoelectrodes commonly generate deep trap states associated with various intrinsic defects such as vacancies, antisites, and dislocations, limiting their photoelectrochemical properties. Herein, it is reported that rhombohedral GaFeO_3_ (GFO) thin‐film photoanodes exhibit defect‐inactive features, which manifest themselves by negligible trap‐states‐associated charge recombination losses during photoelectrochemical water splitting. Unlike conventional defect‐tolerant semiconductors, the origin of the defect‐inactivity in GFO is the strongly preferred antisite formation, suppressing the generation of other defects that act as deep traps. In addition, defect‐inactive GFO films possess really appropriate oxygen vacancy concentration for the oxygen evolution reaction (OER). As a result, the as‐prepared GFO films achieve the surface charge transfer efficiency (*η*_surface_) of 95.1% for photoelectrochemical water splitting at 1.23 V versus RHE without any further modification, which is the highest *η*_surface_ reported of any pristine inorganic photoanodes. The onset potential toward the OER remarkably coincides with the flat band potential of 0.43 V versus RHE. This work not only demonstrates a new benchmark for the surface charge transfer yields of pristine metal oxides for solar water splitting but also enriches the arguments for defect tolerance and highlights the importance of rational tuning of oxygen vacancies.

## Graphical abstract

Semiconductor surface commonly exhibits poor charge transfer for solar water splitting, which requires surface modification such as catalysis loading to enhance water redox kinetics. This article demonstrates that pristine rhombohedral GaFeO_3_ photoanodes without any further modification exhibit a record hole‐transfer efficiency for the oxygen evolution reaction at 1.23 V versus RHE.

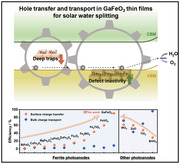

## 1 Introduction

Photoelectrochemical (PEC) water splitting has received widespread attention as one of the promising routes for hydrogen production without carbon footprints.^[^ ^1^, ^2^ ^]^ During PEC water splitting, semiconductor photoelectrodes powered by solar energy generate electron‐hole pairs, and these photoinduced carriers participate in the water oxidation reaction and the water reduction reaction at the semiconductor‐electrolyte interface, releasing O_2_ and H_2_, respectively.^[^ ^3^ ^]^ To achieve a viable and scalable PEC water‐splitting technology, photoelectrodes are required to possess many characteristics, including low cost, good solar‐harvesting capability, suitable band alignment, efficient charge separation and injection, and high chemical stability in aqueous solutions.^[^ ^4^, ^5^, ^6^ ^]^ In this case, iron‐based metal oxides have been considered as attractive photoelectrode materials for PEC water splitting.^[^ ^7^, ^8^ ^]^ Most ferrite materials, such as Fe_2_O_3_,^[^ ^9^ ^]^ ZnFe_2_O_4_,^[^ ^10^ ^]^ BiFeO_3_,^[^ ^11^ ^]^ and LaFeO_3_,^[^ ^12^ ^]^ are Earth‐abundant and dimensionally stable. In addition, they usually exhibit the bandgap energies of 2–2.7 eV, ensuring absorption of visible light.^[^ ^7^ ^]^

Gallium iron oxide, GaFeO_3_ (GFO), is a relatively new ferrite photoanode for solar water splitting, though the ferroelectric‐related properties of GFO have been extensively studied.^[^ ^13^, ^14^ ^]^ Dhanasekaran and co‐workers reported that visible‐light‐responsive GFO nanoparticles can generate H_2_ from water in powder‐based photocatalyst systems.^[^ ^15^ ^]^ The PEC performance of polycrystalline orthorhombic GFO thin films prepared by sol–gel methods are described by Sun et al. in 2020, demonstrating that GFO thin films can promote the water oxidation reaction at the flat band potential.^[^ ^16^ ^]^ It has been reported that the crystal structure of GFO is dependent on the synthesis methods, and can be transformed from orthorhombic to rhombohedral by facile ball‐milling.^[^ ^16^, ^17^, ^18^ ^]^ Moreover, GFO shows significant cation disorder (the exchange of positions between Ga^3+^ and Fe^3+^) due to the similar cation radius, which enables GFO to exhibit unusual electronic structures.^[^ ^19^, ^20^ ^]^ Therefore, it could be interesting to explore the PEC performance of GFO with different phase structures and the relationship between PEC activity and electronic structure.

In this work, for the first time, the PEC properties of rhombohedral GFO thin films with R3c symmetry for solar water splitting are investigated. The novel nanostructured GFO films were prepared by hydrothermal methods, exhibiting *n*‐type semiconductor conductivity, complex optical transitions with a direct bandgap energy of 2.26 eV, hole‐diffusion lengths of ≈14.2 nm, ionic properties with low dispersion in band edges, and strongly preferred antisite formation. Surprisingly, the as‐grown GFO films show the surface charge transfer efficiency of up to 95.1% toward the oxygen evolution reaction at 1.23 V versus RHE, which is the largest value reported for any pristine *n*‐type metal oxides (without surface modification). In addition, the photocurrent onset potential of as‐grown GFO films for PEC water splitting also coincides with the flat band potential, a property rarely seen in inorganic *n*‐type materials. We studied the structure‐performance relationships by experimental investigations combined with DFT calculations, revealing that these impressive and unique PEC properties of GFO films can be attributed to the strong antisite formation between Ga^3+^ and Fe^3+^ as well as the appropriate concentration of oxygen vacancies.

## 2 Results and Discussion

**Figure 1:**
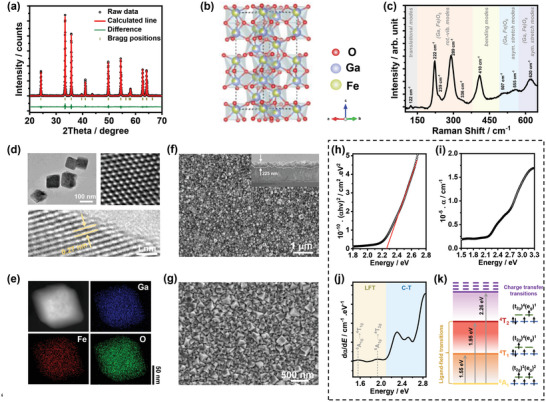
a) XRD of GFO with the Rietveld refinement; b) schematic of the rhombohedral GFO unit cell; c) Raman spectrum of GFO films; d) high‐ and low‐resolution TEM images of GFO nanoparticles; e) EDX mappings of GFO nanoparticles; f,g) top view and cross sectional SEM images of GFO films; h) Tauc plot of GFO films; i) absorption coefficient spectrum of GFO films; j) photon energy differential plot of the absorption coefficient; k) Tanabe–Sugano diagram for Fe^3+^ in octahedral field.

**Figure** **1**a illustrates the powder X‐ray diffraction (XRD) pattern of hydrothermally prepared GFO after annealing at 600 °C (see Supporting Information for details on the synthesis methods), closely matching the diffractogram of rhombohedral GFO previously reported.^[^ ^17^, ^21^, ^22^ ^]^ The Rietveld refinement of the observed pattern further confirms phase‐pure GFO with the rhombohedral structure (R3c space group). The flat green residual curve indicates the accurate fitting, with the statistical correlation parameters of *R*_p_ = 6.56% and *R*_wp_ = 4.91%. The lattice parameters are a = b = 5.0223 (8) Å, c = 13.6286 (1) Å, and volume = 297.72 (6) Å^3^. Figure S1 (Supporting Information) contrasts the XRD patterns of GFO prior to and after annealing treatment, indicating that uncalcined GFO possesses more diffraction peaks. Those extra peaks are assigned to orthorhombic GFO since as‐received GFO prepared by hydrothermal methods can exhibit a mixture of orthorhombic and rhombohedral phases at room temperature.^[^ ^17^, ^21^ ^]^ The following content focuses on the investigation of phase‐pure rhombohedral GFO obtained after annealing at 600 °C. The unit cell of as‐prepared GFO shown in Figure 1b demonstrates that gallium and iron are in octahedral coordination with oxygen. More importantly, it can be observed that atoms in the obtained GFO unit cell are composed of both Ga (blue) and Fe (yellow), indicating the mixed occupancy features of cation sites in GFO. This phenomenon occurs because the similar ionic radius between Ga^3+^ (0.62 Å) and Fe^3+^ (0.64 Å) enables two cations to exchange their sites easily;^[^ ^19^, ^20^ ^]^ consequently, the formation of Ga‐Fe dislocation/disorder, as well as Ga_Fe_ and Fe_Ga_ antisites, is expected in GFO. Although this unusual crystal structure has been commonly seen in GFO, its role in the PEC properties will be discussed further below for the first time.

Figure 1c shows the Raman spectrum of rhombohedral GFO films in the range of 100–650 cm^−1^ under the excitation of 532 nm. Although only 9 Raman signals of the 13 active modes expected for 6 formula units can be observed, the Raman responses are in good agreement with phase‐pure rhombohedral GFO in the literature.^[^ ^18^ ^]^ Figure 1d displays low‐ and high‐resolution transmission electron microscopy (TEM) images of GFO nanoparticles. The clear lattice fringes in the high‐resolution TEM image indicate the crystalline characteristic of GFO samples. The estimated d‐spacing is 0.27 nm, which corresponds to the (104) planes of GFO. The enlarged high‐resolution TEM image (top right) shows regularly arranged bright spots and no one missing in the window, in agreement with the earlier observation, which implies cation vacancies are difficult to form in GFO.^[^ ^23^ ^]^ Energy‐dispersive X‐ray (EDX) analysis of the nanoparticles (Figure 1e) illustrates the homogeneous distribution of Ga, Fe, and O, with a Fe:Ga ratio of 1.08. Scanning electron microscopy (SEM) images of GFO films at two different magnifications in Figure 1f,g demonstrate that nanocrystals cover the FTO substrates entirely and compactly. The cross sectional SEM image shown in the inset reveals the thickness of nanostructured GFO films is ≈225 nm.

The optical properties of GFO films obtained from UV–vis absorption spectra are displayed in Figure 1h–k. The Tauc plot (Figure 1h) reveals a direct bandgap energy of 2.26 eV, which suggests GFO films can harvest the visible light for solar water splitting. We also constructed an indirect Tauc plot for GFO films in Figure S2 (Supporting Information), showing that GFO films also possess an indirect bandgap of 1.84 eV. However, direct band‐band transitions are considered to be predominant in GFO films, as suggested by the following computational studies and the previous study.^[^ ^21^ ^]^ The absorption coefficient (*α*) spectrum in Figure 1i indicates GFO films have strong light‐capture capabilities over the range with the photon energies above the optical bandgap. On the other hand, the *α* spectrum exhibits various optical transitions in the range of 1.5–3.3 eV. The photon energy differential plot of the *α* values in Figure 1j is used to probe these optical features, demonstrating that they are associated with ligand‐field transitions (LFT, yellow area) or charge‐transfer transitions (C‐T, blue area).^[^ ^13^, ^24^ ^]^ As shown below, the band edges of rhombohedral GFO are mainly composed of Fe 3d and O 2p orbitals; therefore, the ligand‐field transitions originate from the FeO_6_ octahedral splitting, including ^6^A_1g_ → ^4^T_1g_ at 1.55 eV and ^6^A_1g_ → ^4^T_2g_ at 1.95 eV.^[^ ^13^ ^]^ The corresponding Tanabe–Sugano diagram is shown in Figure 1k, describing the electron configuration of states linked to the optical transitions.^[^ ^25^ ^]^ The obvious d‐d forbidden transitions further confirm the presence of cation dislocation in GFO films.

**Figure 2:**
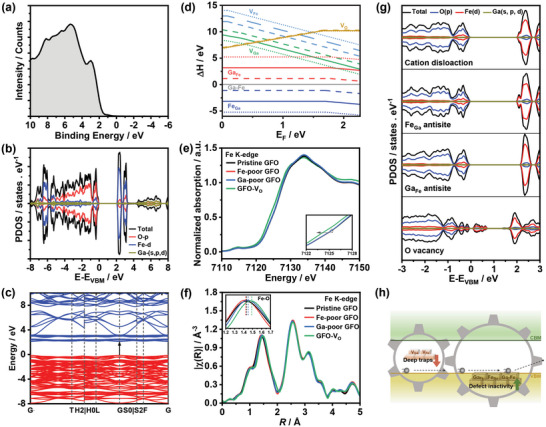
a) VB‐XPS spectrum of GFO films; b) PDOS of rhombohedral GFO; c) corresponding band structure; d) defect formation energies of various intrinsic defects in GFO as a function of the Fermi level at M_0–2_ points; e) normalized XANES spectra of Fe K‐edge; f) corresponding EXAFS spectra; g) PDOS of GFO with intrinsic defects; h) schematic of the effect of highly preferred antisite formation on charge transfer linked to PEC performance.

**Figure** **2**a displays the valence band spectrum of GFO films obtained from XPS measurements (VB‐XPS), which can be rationalized by density functional theory (DFT) calculations. In principle, the projected density of states (PDOS) of rhombohedral GFO in Figure 2b should reproduce the main features of the VB‐XPS. However, the computational results cannot completely match the VB‐XPS. This phenomenon is attributed to the fact that the cross section of the states in valence band is too small to resolve the finer orbital characteristics under the X‐ray excitation. Additionally, the structure used to calculate the PDOS shown in Figure 2b does not contain any defects, but defects are known to have effects on the valence band composition distribution. In this case, the obtained VB‐XPS signals match better the PDOS of GFO with point defects such as oxygen vacancies, as shown below. The PDOS plot reveals that the valence band maximum (VBM) and conduction band minimum (CBM) of GFO is dominated by O 2p and Fe 3d, respectively. Additionally, it can be seen that there is a low degree of hybridization between Ga^3+^ cations and FeO_6_ anion groups, reflecting the ionic feature of GFO. The corresponding band diagram (Figure 2c) illustrates a direct bandgap value of 2.27 eV at G *k*‐point. The rather flat VBM and CBM (low dispersion in the band edges) suggest high effective masses of charge carriers (1.8 m_0_ for holes and 0.96 m_0_ for electrons), which further indicates low carrier mobilities.^[^ ^16^, ^26^ ^]^

In order to study the effects of intrinsic defects on GFO electronic structure, the chemical potential ranges for equilibrium formation of GFO are first constructed in Figure S3 (Supporting Information). As the defect formation energies are dependent on the chemical potential values, five points are chosen in Figure S3 (Supporting Information). These points represent different conditions, cation‐poor/oxygen‐rich (A), cation‐rich/oxygen‐poor (B), and moderate cation and oxygen (M_0–2_). The corresponding chemical potentials of each constituent element are listed in Table S1 (Supporting Information). Figure 2d demonstrates the formation energies of charged defects, including gallium vacancies (V_Ga_), iron vacancies (V_Fe_), oxygen vacancies (V_O_), iron on gallium‐site (Fe_Ga_), gallium on iron‐site (Ga_Fe_), and the exchange of gallium and iron (Ga‐Fe), as a function of the Fermi level at M_0–2_ points. The data show that the generation of cation antisites and dislocation is much easier than cation vacancies. Even under extreme conditions at A and B points, this trend still remains the same, as illustrated in Figure S4 (Supporting Information). Furthermore, at the same oxygen chemical potential of M_0_ point (solid line), Ga‐poor/Fe‐rich (M_1_, dot line) and Fe‐poor/Ga‐rich (M_2_, dash line) conditions also demonstrate the highly preferred antisite formation, though there is a decrease in the defect formation energies of the corresponding cation vacancies.

The GFO electronic structure was then experimentally investigated using X‐ray absorption spectroscopy (XAS). Figure 2e contrasts the normalized Fe K‐edges of the X‐ray absorption near‐edge structure spectra (XANES) of various GFO nanoparticles. It can be seen that the Fe edge energies of cation‐poor GFO do not show any shift compared with pristine GFO, indicating the Fe oxidation states are constant prior to and after physically introducing cation vacancies. This behavior is different from other ferrites, as iron in ferrites is involved in the charge compensation process in the presence of cation or oxygen deficiency, leading to changes in the oxidation state of Fe.^[^ ^27^, ^28^ ^]^ As a result, the data shown in Figure 2e further suggest the highly preferred formation of antisites. For example, in Ga‐poor GFO, Ga‐missing sites are much more favored to be occupied by excessive Fe to form Fe_Ga_ antisites rather than directly from Ga vacancies, which results in unchanged Fe oxidation states. Additionally, we also do observe a clear decrease in the Fe edge energies of GFO with oxygen vacancies (GFO‐V_O_, GFO treated under Ar atmosphere for 3 h). The k^2^‐weighted Fourier transformed extended X‐ray absorption fine structure (EXAFS) spectra of Fe K‐edge in Figure 2f illustrate the complex evolution of scattering. The inset contrasts the Fe—O coordination shell, demonstrating an increase in bond distance upon the increase of Fe content. This observation is another evidence for the generation of cation antisites instead of vacancies when varying the cation ratio, as proposed by Basu et al.^[^ ^29^ ^]^ The increase in the Fe—O bond distance of GFO‐V_O_ is attributed to the weakening of Fe—O covalent bond, which is discussed below.

According to the experimentally obtained range of Fermi levels and the calculated defect formation energies, the PDOS of GFO with various charged or neutral defects were calculated employing DFT+U model. As shown in Figure 2g, GFO with cation dislocation, Fe_Ga_, and Ga_Fe_ antisites do not generate any defect states or deep trap states within the bandgap, which is consistent with previous reports.^[^ ^30^, ^31^, ^32^ ^]^ However, GFO with oxygen vacancies exhibits a wide range of defect states. Similar behavior can also be observed in GFO with cation vacancies, as illustrated in Figure S5 (Supporting Information). Indeed, our recent work demonstrated that cation vacancies lead to various trap states in iron‐based perovskite thin films as well.^[^ ^27^ ^]^ Figure 2h condenses key observations linked to the PEC performance of GFO from the experimental and computational investigations in Figure 2. It can be concluded that the highly preferred Fe_Ga_ and Ga_Fe_ antisite formation inhibits the generation of cation vacancies that act as deep traps, which enables GFO to exhibit defect‐inactive features, thereby reducing trap‐related carrier recombination losses.

**Figure 3:**
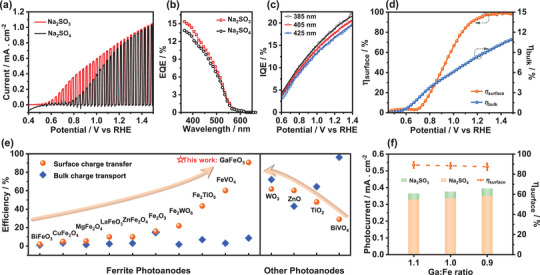
a) LSV curves of pristine GFO films under chopped illumination; b) EQE spectra of GFO films; c) IQE of GFO films obtained from experiments (symbols) and predicted by Gartner equation (lines) at 385, 405, and 425 nm as a function of the potential; d) charge transfer and transport efficiency of GFO films; e) comparison of charge transfer and transport yields between pristine GFO and other metal oxide photoanodes; f) photocurrent density and surface charge transfer efficiency of GFO films with different cation ratios at 1.23 V versus RHE.

**Figure** **3**a describes the photocurrent responses of GFO films in Ar‐saturated 0.1 m Na_2_SO_4_ (black curve) and Na_2_SO_3_ (red curve) electrolytes at pH 12 under the perturbation of square sunlight (AM 1.5G, 100 mW cm^−2^). Although the presence of SO_3_^2−^ enhances the photocurrent density due to the fast kinetics of sulfite oxidation, the photocurrent responses for the water oxidation reaction do not show obvious features of surface charge recombination (e.g., large displacement current in the light on‐transients and off‐transients). The photocurrent onset potentials (defined as the potential at which the photocurrent just appears (over 15 µA cm^−2^) toward sulfite oxidation and oxygen evolution are 0.42 and 0.44 V versus RHE, respectively. On the other hand, according to the optical constant in Figure 1i, the penetration depth of light with photon energy close to the bandgap (2.26 eV) is estimated to be ≈300 nm, though this value should be considered cautiously as the effective film thickness is uncertain when calculating *α* (see Experimental Section). As a result, increasing the thickness of as‐received 225 nm GFO film can further increase carrier generation flux, thereby improving photocurrent responses. Unfortunately, based on the current film preparation routes, we cannot increase GFO film thickness with high quality by simple methods such as increasing precursor concentration and prolonging hydrothermal reaction time.

Figure 3b illustrates the external quantum efficiency (EQE)−wavelength plots of GFO films at 1.23 V versus RHE. The as‐grown GFO photoanodes exhibit significantly larger EQE values than GFO thin films prepared by sol–gel routes across the whole wavelength window.^[^ ^16^ ^]^ For example, the obtained EQE for oxygen evolution at a 365 nm wavelength is more than five times higher than previously reported values. The Tauc representation (Figure S6, Supporting Information) acquired from the EQE spectrum emphasizes that only the photon energies above ≈2.29 eV can excite useful charge carriers to drive the PEC reactions, which is consistent with the optical analysis (Figure 1h). The small tail of EQE spectrum at wavelength above 550 nm can be associated with the contribution of indirect bandgap and oxygen vacancy‐related band tailing in GFO films.^[^ ^33^ ^]^ Figure 3c shows the potential dependence of internal quantum efficiency (IQE) at three wavelengths in the presence of SO_3_^2−^. Based on the Gartner model (see Supporting Information),^[^ ^34^ ^]^ these curves can be employed to estimate the minority carrier (holes) diffusion length (*L*_n_) of GFO films. The IQE–potential plots predicted by Gartner equation agree well with the experimental results, indicating the negligible surface recombination, which is the prerequisite for calculating *L*_n_ from original Gartner expression. From these analyses, the *L*_n_ of GFO films is determined to be as short as 14.2 ± 0.5 nm.

Figure 3d demonstrates the surface charge transfer efficiency (*η*_surface_) and bulk charge transport efficiency (*η*_bulk_) of pristine GFO films as a function of the applied potential. Surprisingly, the *η*_surface_ can reach a value as high as 95.1% at 1.23 V versus RHE. The pH of electrolyte has an impact on the PEC performance of semiconductors, for example, the photocurrent of GFO films decreases under neutral conditions (0.1 m Na_2_SO_4_ or 0.1 m Na_2_SO_3_/NaH_2_PO_4_ aqueous solution), as shown in Figure S7a (Supporting Information). This behavior can be rationalized in terms of the strong coupling effects between hydroxide ions and photogenerated holes, as proposed by Zheng et al.^[^ ^35^ ^]^ Specifically, higher concentrations of hydroxide ions can promote the accumulation of holes at photoanode‐electrolyte interfaces for redox reactions, thereby increasing photocurrent density. On the other hand, the *η*_surface_ calculated using the photocurrent responses under neutral conditions in Figure S7b (Supporting Information) does not exhibit significant changes (e.g., ≈94.7% at 1.23 V vs RHE). This is expected, as the coupling effect of hydroxide ions‐holes at the GFO surface is excluded when extracting photocurrents for *η*_surface_ calculation at the same pH of Na_2_SO_4_ and Na_2_SO_3_ electrolytes, regardless of pH 7 or 12. Thus, our results related to *η*_surface_ can reflect the interface hole‐transfer properties that originate from GFO itself.

Figure 3e summarizes the charge‐transfer performance of pristine *n*‐type metal oxides at 1.23 V versus RHE under 1 sun illumination, which is adopted from the literature.^[^ ^10^, ^11^, ^36^, ^37^, ^38^, ^39^, ^40^, ^41^, ^42^, ^43^, ^44^, ^45^, ^46^ ^]^ It is noted that these values may fluctuate within small ranges depending on the selection of literature. To the best of our knowledge, the as‐prepared GFO films show the highest *η*_surface_ among all the reported pristine inorganic photoanodes (without surface modification). This remarkable and unique behavior can be rationalized in terms of the high defect inactivity in GFO, which significantly ensures the efficient interfacial charge carrier transfer, as discussed above. On the other hand, the *η*_surface_ of GFO films is not high at lower potentials. When the applied potentials are close to the conduction band edge of photoanodes, band bending at semiconductor‐electrolyte interface is weakened, thereby reducing the assistance for carrier separation and transfer. In this case, considering the rather low mobility of electrons in GFO (shown in Figure 2c), it can be expected that a large number of photogenerated electrons tend to be accumulated at the surface of GFO films. Furthermore, poor surface catalytic ability (water oxidation kinetics) is a common phenomenon for most solar absorbers, especially for narrow‐bandgap semiconductors with less positive valence band position, which lowers hole‐transfer rate constant.^[^ ^3^ ^]^ As a result, these surface‐enriched electrons and holes will recombine, resulting in the decrease of *η*_surface_ at lower potentials, even if the concentration of surface traps is low. As for the *η*_bulk_ of GFO films, the values are below 11% across the entire potential window in Figure 3d, though the high defect inactivity of GFO films can also suppress bulk charge recombination associated with trap states. Indeed, the poor bulk charge transport is a general issue in iron‐based absorbers, as displayed in Figure 3e (blue symbol). As confirmed in Figures 2c and 3c, bulk recombination losses arising from slow majority carrier mobilities and short hole‐diffusion length are the main reason for the low *η*_bulk_ of GFO films.

Figure 3f demonstrates the evolution of PEC behavior upon varying Ga:Fe ratios at 1.23 V versus RHE. The photocurrent density is adopted from linear sweep voltammograms (LSV) shown in Figure S8 (Supporting Information). The SEM images in Figure S9 (Supporting Information) contrast the morphologies of three films, indicating that the particle size of GFO gradually increases with Fe content. This leads to large variations in the thickness of films directly grown by hydrothermal methods. For example, when Ga:Fe ratio is slightly below 0.9 in the hydrothermal solution, we found no trace of GFO growth on FTO substrates. Thus, for the sake of obtaining comparable PEC results, these GFO films were prepared by spin‐coating nanoparticle suspension onto FTO substrates. Although the thickness of GFO films generated by spin coating is also affected by nanoparticle sizes, this method ensures that a comparable number of GFO nanoparticles are deposited on the FTO substrate. The XRD patterns in Figure S10 (Supporting Information) show the structure of GFO does not change within the selected composition range, which agrees with previous reports.^[^ ^16^, ^22^ ^]^ The data (Figure 3f) show that the photocurrents increase with decreasing Ga:Fe ratios. One of the origins of this phenomenon is the narrowing of the bandgap energies with the increase of Fe content, as shown in Figure S11 (Supporting Information). Although the observed trend in photocurrent responses is consistent with the earlier study,^[^ ^16^ ^]^ caution is herein required when obtaining the specific difference in photocurrent densities between GFO films with different cation ratios due to the different film thickness. It can also be seen that the *η*_surface_ of three GFO films for the oxygen evolution reaction (OER) is almost at the same level. Furthermore, the transient photocurrent responses (Figure S12, Supporting Information) of GFO films are in‐phase with the perturbation of light and they do not show obvious change upon varying cation composition. Therefore, these results further confirm that surface and bulk charge recombination linked to trap states is very less in GFO films due to the formation of strongly preferred antisites.^[^ ^27^, ^47^ ^]^

**Figure 4:**
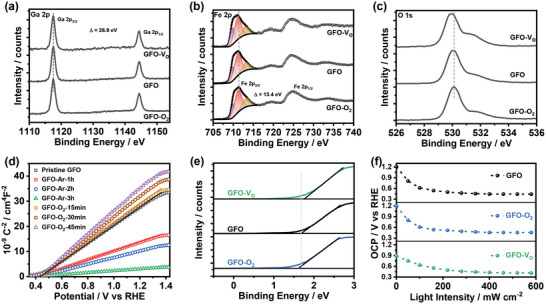
Changes in the properties of GFO films upon Ar and O_2_ treatment: a) XPS spectra of Ga 2p; b) XPS spectra of Fe 2p; c) XPS spectra of O1s; d) Mott–Schottky plots; e) VB‐XPS spectra; f) open circuit potential as a function of the light intensity in O_2_‐saturated 0.1 m Na_2_SO_4_ aqueous solutions at pH 12.

Next, we investigated another intrinsic defect, oxygen vacancy, that has significant impacts on the PEC performance of photoanodes. Thermal reduction and oxidation were used to generate various GFO films with different oxygen vacancy content (see Experimental Section). GFO films re‐calcined under Ar for 3 h (GFO‐V_O_) and O_2_ for 45 min (GFO‐O_2_) do not display evident changes in morphology and optical properties, as shown in Figures S13 and S14 (Supporting Information), respectively. The surface composition information of various GFO films was acquired by X‐ray photoelectron spectroscopy (XPS). The survey spectra (Figure S15, Supporting Information) exhibit various characteristics, including Ga, Fe, O, and C photoemission peaks and their Auger signals. As a first approximation, the Ga cation is in a + 3 oxidation state, which is verified by the photoemission positions of Ga 2p_3/2_ at 1117.5–1117.9 eV and Ga 2p_1/2_ at 1144.4–1144.8 eV in **Figure** **4**a, with a spin–orbit splitting between the doublet (Δ) of ≈26.9 eV.^[^ ^16^ ^]^ As for the Fe 2p region (Figure 4b), it contains four photoemission responses, with two larger components being associated with Fe 2p_3/2_ at 711.2 eV and Fe 2p_1/2_ at 724.6 eV. The reset two components are their corresponding satellite peaks at ≈719.4 and 732.8 eV. These features highlight the dominance of Fe cation with a + 3 oxidation state in the lattice.^[^ ^48^, ^49^ ^]^ To semi‐quantitatively analyze the proportion of different Fe species, photoemission deconvolution is a direct approach. However, the complex feature of Fe 2p signal induced by multiple‐splitting, multiple oxidation states, and charge transfer effects increases the difficulty and inaccuracy of Fe 2p deconvolution.^[^ ^16^, ^48^ ^]^ In this case, we implemented a straightforward approximation according to previous studies on Fe 2p photoemission.^[^ ^28^, ^49^ ^]^ Fe core‐level is deconvoluted to three peaks assigned to Fe^2+^ (purple), Fe^3+^ (red), and Fe^4+^ (yellow). As displayed in Table S2 (Supporting Information), GFO‐V_O_ exhibits the maximum Fe^2+^ content and the minimum Fe^4+^ content (the decrease of Fe oxidation states), which agrees well with the XANES results (Figure 2e). This phenomenon indicates that oxygen vacancies are introduced into GFO films upon annealing under Ar atmosphere, as oxygen vacancies can compensate for the reduction of Fe ions to maintain charge neutrality. In the O 1s region (Figure 4c), the predominant peak at 530 eV corresponds to the oxygen in the lattice, while the broad peak at higher binding energies can be assigned to surface hydroxyl, carbonylated species, and absorbed water.^[^ ^50^ ^]^ The intensity of the broad peak gradually grows from thermal oxidation to thermal reduction. Previous research usually attributed this observation to the effect of increased oxygen vacancies;^[^ ^28^, ^51^ ^]^ however, Idriss proposed a different view that changes in O 1s signals cannot be linked to the role of oxygen vacancies, as it is impossible for an absent atom to exhibit XPS signals.^[^ ^52^ ^]^ We then conducted electron paramagnetic resonance (EPR) characterizations at room temperature to analyze the trend in oxygen vacancies. As displayed in Figure S16 (Supporting Information), the EPR signal becomes stronger as the heat treatment atmosphere changes from oxidizing to reducing conditions, suggesting an increase in the oxygen vacancy content (GFO‐O_2_ < GFO < GFO‐V_O_).

As a direct observation of the XPS data, the Ga 2p photoemissions remain almost at the same position, while the Fe 2p and O 1s photoemissions shift toward low binding energies (BE) direction with decreasing O_2_ content in the calcination atmosphere. Moreover, the quantitative refinement of XRD patterns (Figure S17, Supporting Information; Figure 1a) reveals that the lattice volume of the GFO unit cell gradually drops, as the annealing atmosphere changes from reduction to oxidation. These phenomena further verify the variation trend of oxygen vacancy content obtained by EPR analysis. As concluded by Sun et al., the changes in Fe oxidation states (induced by oxygen vacancy) can manifest the changes in lattice volume and Fe—O covalent bond,^[^ ^48^ ^]^ for example, the increase of Fe^2+^ content can expand the unit cell and lower the BE of both Fe 2p and O 1s. It should be noted that the position of Ga 2p signals does not show obvious shift, which may be determined by multiple factors such as oxygen vacancy content and antisite status after re‐heat treatment. Further studies to understand the thermal temperature dependence of antisite status and the effect of oxygen vacancies on Ga^3+^ in GFO unit cells will help to further rationalize this phenomenon. The surface ratio of Fe:Ga estimated from the XPS spectrum is ≈1.05, which is close to the bulk cation ratio acquired from the EDX analysis.

Figure 4d shows the Mott–Schottky plots of pristine, Ar‐treated, and O_2_‐treated GFO films measured at 1 kHz in 0.1 m Na_2_SO_4_ aqueous solutions at pH 12. The positive slopes of the linear regions are the indicative of the *n*‐type semiconductor conductivity. The majority carrier density (*N*_d_) of various GFO films is summarized in Table S3 (Supporting Information) based on the Mott–Schottky equation. The data show that Ar treatment results in the increase of the *N*_d_, while O_2_ treatment possesses opposite effects. Figure S18 (Supporting Information) contrasts the Mott–Schottky plots of GFO, GFO‐V_O_, and GFO‐O_2_ films at different frequencies, demonstrating that the slope of the plots varies with frequency. This phenomenon can be rationalized in terms of the frequency dependence of the dielectric constant. In addition, there is uncertainty in the effective surface area of GFO films. Therefore, although the observed trend in *N*_d_ upon Ar and O_2_ treatment is clear and unambiguous, the absolute values estimated from Mott–Schottky equation must be considered cautiously. The pristine GFO exhibits a flat band potential (*U*_fb_) of 0.43 V versus RHE. It is worth mentioning that the photocurrent onset potential of GFO films for the water oxidation reaction (Figure 3a) is very close to the estimated *U*_fb_. Upon Ar treatment, the *U*_fb_ shift toward negative potential direction, which agrees with the increase in the electron concentration. As for O_2_‐treated GFO films, the *U*_fb_ remains almost unchanged under O_2_ flow for 15 min but gradually shifts toward positive potential direction with increasing treatment time. The VB‐XPS spectrum of various GFO films (Figure 4e) further demonstrates that the introduction of oxygen vacancies enables the Fermi level of GFO to rise relative to the valence band edge position. Although the observed trend in *U*_fb_ seems reasonable, the determination of *U*_fb_ using Mott–Schottky equation is full of uncertainty, as systemically reported by Hankin et al.^[^ ^53^ ^]^ In this case, the *U*_fb_ of GFO films was further confirmed by the light‐saturated OCP method, that is, the open circuit potential (OCP) of electrode is measured under high irradiance. As shown in Figure 4f, the light‐saturated OCP of GFO films is ≈0.44 V versus RHE, indicating that the *U*_fb_ of GFO obtained by the light‐saturated OCP method is highly close to that estimated from the Mott–Schottky equation. Comparing the *U*_fb_ of GFO‐O_2_ films acquired by these two methods, we can also see the identical phenomenon. Thus, the consistency of two independent data sets increases the reliability of the obtained *U*_fb_ value of GFO and GFO‐O_2_ films. However, as for the *U*_fb_ of GFO‐V_O_ films, two methods exhibit large differences, i.e., 0.41 V versus RHE and 0.35 V versus RHE are determined from the light‐saturated OCP and the Mott–Schottky plot, respectively. The latter shows more negative *U*_fb_, which can be attributed to the complex contribution of the Helmholtz layer after the introduction of oxygen vacancies that can generate defect states and the underestimation of film surface area.^[^ ^53^ ^]^

Another notable feature in Figure 4d is that the linear region generated by pristine and O_2_‐treated GFO films can be extrapolated over a wide potential window (near 1 V range), which tends to indicate very few sub‐bandgap states below the CBM.^[^ ^16^, ^48^ ^]^ This statement can be further confirmed by the OCP of GFO photoanodes in the dark (the OCP values at zero irradiance in Figure 4f). The dark OCP of GFO and GFO‐O_2_ films are ideally close to the redox potential of the OER reaction (1.23 V vs RHE), strongly suggesting no Fermi‐level pinning. However, GFO‐V_O_ films exhibit a more negative dark OCP value, which indicates the presence of Fermi‐level pinning induced by oxygen‐vacancy‐related defect states. In addition, solution pH value dependence of *U*_fb_ in Figure S19 (Supporting Information) shows that the *U*_fb_ of GFO, GFO‐O_2,_ and GFO‐V_O_ changes by ≈−0.059, −0.059, and −0.047 V, respectively, for each pH unit increase. Typically, the linear *U*_fb_‐pH plot with a slope of −0.059 is an indication of the absence of Fermi level pinning, whereas the slower change of *U*_fb_ as a function of the pH value is considered as the effect of Fermi level pinning.^[^ ^54^, ^55^ ^]^ Therefore, two independent results highlight a rather low density of defect states (no Fermi level pinning) below the CBM of GFO and GFO‐O_2_ films, which is consistent with the characteristic of high defect inactivity in GFO proposed above. However, excessive oxygen vacancies introduced by Ar treatment can produce trap states, resulting in Fermi‐level pinning in GFO‐V_O_ films.

**Figure 5:**
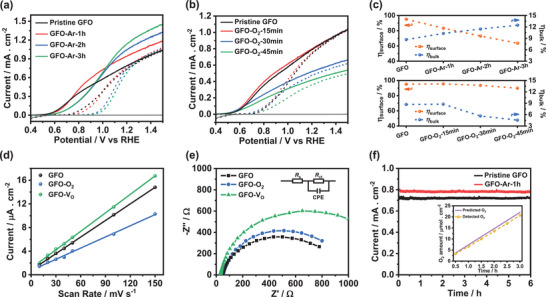
a) photocurrent responses of GFO films annealed in Ar for different times in Ar‐purged 0.1 m Na_2_SO_3_ (solid line) and Na_2_SO_4_ (dash line) aqueous solutions at pH 12; b) identical measurement for O_2_‐treated GFO films; c) corresponding *η*_bulk_ and *η*_surface_ of various GFO films as a function of the thermal reduction and oxidation time at 1.23 V versus RHE; d) scan rate dependence of current density; e) EIS curves of three GFO films under 1‐sun illumination; f) stability test for GFO and GFO‐Ar‐1 h films and predicted and detected oxygen generation amount of GFO‐Ar‐1 h films at 1.23 V versus RHE in the inset. The Ar and O_2_‐treated GFO films are labeled GFO‐Ar or O_2_‐x, where x is the Ar or O_2_ treatment time.

LSV curves of Ar‐treated and O_2_‐treated GFO films were recorded in Ar‐purged 0.1 m Na_2_SO_3_ (solid line) and Na_2_SO_4_ (dash line) aqueous solutions at pH 12 under continuous AM 1.5G illumination. The photocurrent density of Ar‐treated GFO films for sulfite oxidation obtains great enhancement cross section with potentials more positive than ≈0.9 V versus RHE, as shown in **Figure** **5**a. Unfortunately, Ar treatment does not obviously improve the photocurrent responses toward oxygen evolution but instead decreases the photocurrent density at potentials below 1.2 V versus RHE. It can also be seen that Ar treatment enables the fill factors of LSV curves to significantly reduce, which indicates more defect states are introduced in GFO films upon Ar treatment.^[^ ^56^, ^57^ ^]^ Indeed, the DFT calculations (Figure 2g) and electrochemical characterization (Figure 4d,f) have emphasized that oxygen vacancies can generate sub‐bandgap states in GFO. As for the photocurrent responses of O_2_‐treated GFO films in Figure 5b, the data reveal that the slight elimination of oxygen vacancy has minimal effects on photocurrent responses, but photocurrent density significantly drops after O_2_ treatment for more than 15 min. In addition, although O_2_ treatment can further decrease the concentration of defects associated with oxygen vacancy, the fill factors of LSV curves for O_2_‐treated GFO films do not exhibit obvious enhancement. This phenomenon can be rationalized in terms of inefficient charge carrier transport at FTO‐GFO interfaces and grain boundaries in the bulk of GFO films.^[^ ^2^ ^]^ Figure 5c summarizes the charge transfer and transport yields of these photoanodes at 1.23 V versus RHE, indicating that the *η*_bulk_ of GFO films gradually increases with the Ar treatment time and decreases with the O_2_ treatment time. The evolution of *η*_bulk_ is linked to the change of *N*_d_ (Figure 4d). The enhanced *N*_d_ can facilitate the charge transport in the bulk, thereby increasing the photocurrent density of Ar‐treated GFO films in the presence of hole scavengers. Conversely, lower *N*_d_ in O_2_‐treated GFO films is the main origin for the reduced photocurrent density.

With regards to the evolution of *η*_surface_, a downward trend can be seen in GFO films upon both thermal oxidation and reduction. The slight decrease of *η*_surface_ from 95.1% to 93.6% after O_2_ treatment for 30 min is ascribed to the reduction of surface oxygen vacancies since it has been proposed that oxygen vacancies are active sites for promoting the oxygen evolution reaction at photoelectrode surface.^[^ ^58^ ^]^ We then employed electrochemically active surface area (ECSA) measurements to assess the active surface area of films. Current densities obtained from cyclic voltammograms (Figure S20, Supporting Information) were plotted as a function of the scan rate, where the slope is proportional to ECSA in Figure 5d. The data show that GFO‐O_2_ has the lowest surface‐active sites, indicating the elimination of oxygen vacancies can lower surface electrocatalytic ability.^[^ ^59^, ^60^ ^]^ Figure S21 (Supporting Information) contrasts the electrochemical water oxidation performance of three films, which further highlights the beneficial impact of oxygen vacancies on the water oxidation reaction. Although GFO‐V_O_ exhibits the best surface electrocatalytic properties, there is an apparent decrease in *η*_surface_ with an increased Ar treatment time. This phenomenon originates from the trap states induced by excessive oxygen vacancies, exacerbating surface charge recombination; therefore, the photocurrent responses of Ar‐treated GFO toward oxygen evolution are lower than pristine GFO, despite the greatly enhanced bulk electron conductivity and surface‐active sites. Electrochemical impedance spectra (EIS) in Figure 5e were conducted to quantitatively analyze the interfacial charge transfer dynamics. To rationalize the EIS data, an equivalent circuit model shown in the inset was constructed, including a series resistance (*R*_s_), a surface charge transfer resistance (*R*_ct_), and a constant phase angle element (CPE).^[^ ^61^, ^62^ ^]^ The fitted *R*_ct_ values of three GFO films are displayed in Table S4 (Supporting Information), further confirming that GFO‐O_2_ and GFO‐V_O_ possess larger surface hole‐transfer barriers than pristine GFO. Based on the discussion above, it can be concluded that oxygen vacancy engineering via thermal reduction in an inert atmosphere can increase *η*_bulk_ at the expense of *η*_surface_. On the other hand, the removal of oxygen vacancies by longer thermal oxidation negatively affects the PEC performance of surface “defect‐free” photoanodes. Yet, the GFO films prepared here possess appropriate amount of oxygen vacancies, ensuring the highly efficient surface hole transfer toward the water oxidation reaction.

The *J–t* plots of pristine GFO and GFO‐Ar‐1 h films in Figure 5f were recorded at 1.23 V versus RHE in Ar‐saturated 0.1 m Na_2_SO_4_ solutions at pH 12 under 1‐sun illumination, indicating the stable PEC performance of GFO photoanodes for solar water splitting. The inset of Figure 5f illustrates the amount of oxygen generation as a function of the time for GFO‐Ar‐1 h films (under the same experimental conditions as the stability test). The detected oxygen evolution amount (symbol) is close to the corresponding predicted amount (line) estimated from the photocurrent density of GFO‐Ar‐1 h (Figure 5f), with the Faradaic efficiencies of ≈90%, confirming that photogenerated electrons in GFO films are mainly used for the water oxidation reaction. We attribute the Faradaic efficiencies not approaching 100% to three possible reasons. 1) There are other side reactions that may occur due to the contamination of electrolyte or film surface. 2) The generated oxygen is not fully detected as a small amount of gas may escape. 3) The highly textured topography of GFO films results in a much larger effective area than the geometric area. The geometric working area of GFO films used for stability tests (predicting oxygen evolution amount) is slightly larger than that used for detecting oxygen yield. On the other hand, we used the geometric area to calculate predicted and detected oxygen production per unit area. In this case, predicted oxygen production is overestimated to a greater extent than detected oxygen yield after area normalization.

## 3 Conclusion

For the first time, nanostructured rhombohedral GFO thin‐film photoanodes were successfully prepared by facile hydrothermal methods, generating stable photocurrent responses with the onset potential close to the *U*_fb_ for the water oxidation reaction. The photocurrent density of GFO‐V_O_ films toward sulfite oxidation reaches 1.21 mA cm^−2^ at 1.23 V versus RHE. Remarkably, the as‐prepared pristine GFO films exhibit surface hole‐transfer efficiency of over 95% for solar water splitting at 1.23 V versus RHE without loading any cocatalysts. Such high *η*_surface_ values of GFO films have surpassed all pristine *n*‐type metal oxides reported so far. We rationalized this remarkable performance from two aspects through integrated experimental and theoretical studies. First, the highly preferred antisite formation in GFO suppresses the generation of trap states originating from cation vacancies, thus reducing trap‐states‐associated charge carrier recombination. This behavior is different from conventional defect‐tolerant semiconductors, which is confirmed by HR‐TEM, DFT calculations, XAS, *J–V* curves, and transit photocurrent responses. Second, the as‐prepared GFO films generate appropriate concentration of oxygen vacancies for solar water splitting, which not only leads to the absence of trap‐states associated with oxygen vacancies at the surface but also provides sufficient reaction kinetics for the water oxidation reaction, as illustrated by the PEC and electrochemical responses of GFO films upon thermal oxidation and reduction. This discovery also emphasizes that the oxygen vacancy engineering via re‐calcining the material in an inert atmosphere can result in a trade‐off issue between *η*_surface_ and *η*_bulk_.

## Conflict of Interest

The authors declare no conflict of interest.

## Supporting information

Supporting Information

## Data Availability

The data that support the findings of this study are available from the corresponding author upon reasonable request.
