# Supplementary material for: Pristine GaFeO3 Photoanodes with Surface Charge Transfer Efficiency of Almost Unity at 1.23 V for Photoelectrochemical Water Splitting
Source: Adv Sci (Weinh). 2023 Jan 19;10(8):2205907. doi: 10.1002/advs.202205907 (PMC10015867; doi:10.1002/advs.202205907)
Supplement: Supplementary file 1 — Supporting Information [file ADVS-10-2205907-s001.pdf]

ADVANCED SCIENCE
Open Access

Supporting Information

for *Adv. Sci.*, DOI 10.1002/advs.202205907

# Pristine $GaFeO_3$ Photoanodes with Surface Charge Transfer Efficiency of Almost Unity at 1.23 V for Photoelectrochemical Water Splitting

*Xin Sun, Min Wang, Hai-Fang Li, Linxing Meng, Xiao-Jun Lv, Liang Li and Meicheng Li**

# Supporting Information

## Pristine $GaFeO_3$ Photoanodes with Surface Charge Transfer Efficiency of almost Unity at 1.23 V for Photoelectrochemical Water Splitting

*Xin Sun, Min Wang, Hai-Fang Li, Linxing Meng, Xiao-Jun Lv, Liang Li and Meicheng Li* *

Dr. Xin Sun, Min Wang, Dr. Hai-Fang Li, Prof. Xiao-Jun Lv, Prof. Meicheng Li
State Key Laboratory of Alternate Electrical Power System with Renewable Energy Sources, School of New Energy, North China Electric Power University, Beijing, 102206, China.
E-mail: mcli@ncepu.edu.cn

Dr. Linxing Meng, Prof. Liang Li
School of Physical Science and Technology, Jiangsu Key Laboratory of Thin Films, Center for Energy Conversion Materials & Physics (CECMP), Soochow University, Suzhou, 215006, China.

**Experimental Section.** GFO films preparation, characterization methods and computational methods.

**S1.** Gartner model for analysis of the photocurrent responses and estimation of the hole diffusion length.

**Figure S1.** XRD patterns of GFO films before and after annealing at 600 $^oC$.

**Figure S2.** Indirect Tauc plot of GFO films.

**Figure S3.** Thermodynamic stable chemical potential ranges for GFO with respect to competing phases.

**Figure S4.** Defect formation energies of various intrinsic defects in GFO as a function of the Fermi level at possible extreme conditions.

**Figure S5.** Projected density of states of rhombohedral of GFO with cation vacancy defects.

**Figure S6.** Tauc plot representation of the EQE spectrum in the presence of $Na_2SO_3$.

**Figure S7.** *J-V* curves of GFO films under neutral conditions and corresponding charge transfer and transport yields.

**Figure S8.** Photocurrent responses of GFO films with different cation ratio.

**Figure S9.** Top view SEM images of nanoparticle-based GFO films with different cation ratio.

**Figure S10.** XRD patterns of GFO with different compositions.

**Figure S11.** Tauc plots of nanoparticle-based GFO films with different cation ratio.

**Figure S12.** Transient photocurrent responses of various GFO films.

**Figure S13.** Top view SEM images of GFO films prior and after thermal reduction and oxidation.

**Figure S14.** UV-vis absorption spectra of GFO, GFO-$V_O$ and GFO-$O_2$ films.

**Figure S15.** Survey XPS spectra of GFO, GFO-$V_O$ and GFO-$O_2$ films.

**Figure S16.** EPR spectra of GFO, GFO-$V_O$ and GFO-$O_2$ films at room temperature.

**Figure S17.** Rietveld refined XRD of GFO after Ar and $O_2$ treatment.

**Figure S18.** Mott-Schottky plots of three GFO films recorded at different frequencies.

**Figure S19.** Solution pH dependence of flat band potential.

**Figure S20.** Cyclic voltammograms of three GFO films recorded at different scan rates.

**Figure S21.** Electrocatalytic performance of GFO, GFO-$V_O$ and GFO-$O_2$ films.

**Table S1.** Chemical potential values of gallium, iron and oxygen at various points.

**Table S2.** Contrast between various Fe species content in pristine GFO, Ar-treated GFO and O2-treated GFO films obtained from the deconvolution of Fe $2p_{3/2}$ peaks.

**Table S3.** The electron density of various GFO films calculated using Mott-Schottky equation.

**Table S4.** The fitting results of electrochemical impedance spectra.

## Experimental Section

**GFO thin films preparation.** Nanostructured rhombohedral GFO thin-film photoanodes were prepared using a hydrothermal method for the first time, which is developed based on the synthesis of the GFO nanoparticles.[1] Stoichiometric $Ga(NO_3)_3·6H_2O$ and $Fe(NO_3)_3·9H_2O$ were dissolved in 15 mL distilled water (18.2 MΩ resistance), and the mixture was further stirred at room temperature for 1 h. Subsequently, the solution was transferred into a 25 mL Teflon-lined autoclave. A piece of F:$SnO_2$ (FTO) substrate with the conductive side facing down was placed in Teflon-vessel as well. The hydrothermal synthesis was then conducted at 250 $^{o}$C for 5 h. After the autoclave was naturally cooled to room temperature, the as-grown films were taken out, followed by washing and drying. Finally, the films were calcined in a muffle furnace at 600 $^{o}$C for 3 h to generate pristine GFO films.

Stoichiometric ratio of gallium and iron salts in precursor solution was changed following $Ga_{2-x}Fe_xO_3$ (x from 0.95 to 1.05) compositions to prepare GFO with different cation ratio. The rest of the procedure is the same as the preparation of pristine GFO films.

Ar-treated GFO films were obtained by re-calcining pristine GFO films in a tube furnace under argon atmosphere (Ar flow rate of 95 cc min$^{-1}$) at 500 $^{o}$C for different durations (1 h, 2 h and 3 h). Similar procedure was conducted to prepare $O_2$-treated GFO films. The thermal oxidation condition was heating at 300 $^{o}$C under oxygen purge (60 cc min$^{-1}$) for various times (15 min, 30 min and 45 min). The Ar and $O_2$-treated GFO films are labeled GFO-Ar or $O_2$-x, where x is the Ar or $O_2$ treatment time.

**Characterization methods.** Powder X-ray diffractions (Bruker D8 Focus) recorded the crystal structure of GFO, using a Cu Kα source (λ=1.54016 Å). Raman spectra were acquired employing a Renishaw machine (inVia-Qontor). The morphology of GFO films was observed by scanning electron microscopy (Hitachi SU8010). Transmission electron microscopy (TEM) images were obtained by a JEM-2100F. For the preparation of TEM samples, GFO nanoparticles were embedded in epoxy resin, and then cut into thin sections using an ultramicrotome (Leica EM UC6). Energy-dispersive X-ray detector (Oxford X-Max-80) coupled to the TEM was employed to depict the element distribution of GFO and quantify cation content. X-ray photoelectron spectroscopy were conducted on a Thermo-Fisher Scientific instrument (ESCALAB 250Xi). X-ray absorption spectra of Fe K-edges were acquired, ex-situ, at beamline BL 14W of Shanghai Synchrotron Radiation Facility. Electron paramagnetic resonance characterizations were carried out on Bruker EMX PLUS. The Shimadzu UV-2600 spectrophotometer was used to obtain the UV-visible absorption spectrum of GFO films. The absorption coefficient ($\alpha$) is estimated from the equation:

$$\alpha = \frac{2.303A}{t}$$

where $A$ is the absorbance, and $t$ is the film thickness. In this work, $t$ value was obtained from the cross-sectional SEM image of GFO films. It should be noted that this apparent film thickness may underestimate the $\alpha$ values due to the porous or textured characteristics of GFO films, though this phenomenon plays minimal effect on the data analysis in the main text. Accurate calculation of the absorption coefficient values requires obtaining the effective film thickness.

All (photo)electrochemical measurements were performed with an IviumStat potentiostat, employing three-electrode configuration (GFO working electrode, carbon counter electrode, and Ag-AgCl (KCl saturated) reference electrode). Linear sweep voltammograms were measured by sweeping the potential to oxidative direction at 5 mV $s^{-1}$ in Ar-purged 0.1 M $Na_2SO_4$ or 0.1 M $Na_2SO_3$ aqueous solutions at pH 12. Electrochemical impedance spectra were performed at frequencies ranging from 0.1 Hz to 100 kHz at the open circuit potential under 1-sun illumination in 0.1 M Ar-saturated $Na_2SO_4$ at pH 12. Mott–Schottky plots were carried out in the dark at frequencies of 0.5, 1.0 and 1.5 kHz with a sinusoidal modulation of 15 mV in 0.1 M Ar-saturated $Na_2SO_4$ (pH 12). The open circuit potential (OCP) was recorded in $O_2$-saturated $Na_2SO_4$ aqueous solutions (in the absence of hole scavengers) at pH 12. All the OCP values were obtained after reaching steady-state (1 h in the dark and 30 min under illumination). Solution pH values were adjusted using concentrated NaOH aqueous solution. 1-sun illumination condition was simulated by a 300 W xenon lamp coupled with an AM 1.5 G filter. The light intensity of 100 mW $cm^{-2}$ was calibrated using a standard Si photodiode.

Other light intensities used in the light-saturated OCP measurement were also determined by the standard Si photodiode. The square wave light perturbation was conducted at a frequency of 3.33 Hz. All measured potentials with respect to the Ag-AgCl reference electrode have been converted to against the reversible hydrogen electrode (RHE) by the Nernst equation:

$$E\ (vs\ RHE)\ =\ E\ (vs\ Ag/AgCl\ )\ +\ E_{Ag/Agcl}\ +\ 0.059\ V\ \ \times\ pH$$

where $E_{\mathrm{Ag/AgCl}}$ equals 0.197 V vs NHE at 25 °C. Surface charge transfer efficiencies ($\eta_{\mathrm{surface}}$) were calculated from the equation:

$$\eta_{\mathrm{surface}}\ =\frac{j_{\mathrm{water\ oxidation}}}{j_{\mathrm{sulfite\ oxidation}}}$$

and bulk charge transport efficiencies ($\eta_{\mathrm{bulk}}$) were extracted from the equation:

$$\eta_{\mathrm{bulk}}\ =\frac{j_{\mathrm{sulfite\ oxidation}}}{j_{\mathrm{theoretical\ maximum}}}$$

where $j_{\mathrm{water\ oxidation}}$ and $j_{\mathrm{sulfite\ oxidation}}$ are the photocurrent density measured in $Na_2SO_4$ and $Na_2SO_3$ aqueous solutions, respectively. $j_{\mathrm{theoretical\ maximum}}$ is the theoretical maximum photocurrent density estimated from the absorptance spectrum of GFO combined with the standard AM 1.5G spectrum. The oxygen evolution amount was acquired by gas chromatography (Shimadzu GC-2014C). Electrochemically active surface area (ECSA) was acquired by carrying out cyclic voltammograms at scan rates between 10 and 150 mV $s^{-1}$. The difference in the cathodic and anodic current densities at 0.85 V vs RHE was plotted as a function of the scan rate. The slope of the linear region can be used to represent ECSA, as the slope is twice of the double layer capacitance:

$$\mathrm{ECSA}\ \propto C=\frac{\mathrm{d}Q/\mathrm{d}t}{\mathrm{d}E/\mathrm{d}t}=\frac{i(E)}{v}$$

where $C$ is the electrochemical capacitance, $i(E)$ is the current density at applied potential $E$, and $v$ is the scan rate.

**Computational methods.** Density functional theory calculations were carried out on the Vienna ab initio simulation software package (VASP 5.4.4) using Perdew–Burke–Ernzerhof (PBE) functional combined with generalized gradient approximation under projector augmented wave (PAW) method.[2-4] A Hubbard U value of 3.5 eV on Fe 3d was applied to address the electron-correlation effects arising from high localization of d-electrons.[5,6] Considering the influence of spin polarization, a 6×6×3 Monkhorst-Pack *k*-point grid was used to optimize the crystal structure of GFO obtained from experiments. The Gaussian broadening is set to σ = 0.05 eV to describe the electron orbital occupation. The convergence accuracy criterion was set at $10^{-5}$ eV for electronic energy and below 0.02 eV/ Å for forces on atoms. The cutoff energy of the plane wave was set to 520 eV in all calculations.

A 2 × 2 × 1 supercell with 120 atoms was employed for all defect calculations to minimize the interactions between defects. Defect formation energies, in charge state *q*

($\Delta H_f^{D,q}$), were calculated using the following equation:

$$\Delta H_f^{D,q} = \left(E^{D,q} - E^{perfect}\right) \pm \sum\{n_i(E_i + \mu_i)\} + q(E_{VBM} + E_F) + E_{corr}$$

where $E^{D,q}$ is the energy of supercell containing a defect, D, in a charge state, $q$; $E^{perfect}$ is the energy of the perfect supercell; $n_i$ is the added or removed atom number when forming the defect; $E_i$ is the reference energy in its standard state of the element; $\mu_i$ is the chemical potential of the defect ($i$ is Ga, Fe and O); $E_{VBM}$ is the energy of the valence band maximum; $E_F$ is the Fermi energy relative to $E_{VBM}$; $E_{corr}$ is a correction to consider the limitations of such supercell defect calculations including Coulombic interactions between defects, spurious electrostatic potential alignment of defect supercell relative to the perfect supercell, and total energy owing to erroneous band filling.

**S1.** Gartner model for analysis of the photocurrent responses and estimation of the hole diffusion length:

The theoretical internal quantum efficiency (IQE) of GFO films as a function of the potential shown in Figure 5e was calculated following the Gartner expression:[7,8]

$$IQE = 1 - \frac{e^{(-\alpha W)}}{1 + \alpha L_{\mathrm{n}}}$$

where, $L_n$ is the hole diffusion length, $\alpha$ is the absorption coefficient, and $W$ is the width of charge space regions calculated as:

$$W = \sqrt{[\frac{2\varepsilon\varepsilon_0}{qN_{\mathrm{D}}}(E - U_{fb})]}$$

where, $U_{\mathrm{fb}}$ is the flat band potential, $N_{\mathrm{D}}$ is the majority carrier density, $e$ is the permittivity of materials, $e_0$ is the dielectric constant of free space, and $E$ is the applied potential.

Based on the above equations, a $L_{\mathrm{n}}$ = 14.2 ± 0.5 nm can be estimated by using the experimental values of $\alpha$, $U_{\mathrm{fb}}$, and $N_{\mathrm{D}}$ over the range of wavelengths.

References:

[1] S. Mukherjee, M. Mishra, P. Swarnakar, S. Sanwlani, S. Dash, A. Roy, Mater. Adv. **2022**, 3, 3980.
[2] G. Kresse, J. Furthmüller, Phys. Rev. B **1996**, 54, 11169.
[3] G. Kresse, J. Furthmüller, Comput. Mater. Sci. **1996**, 6, 15.
[4] J. P. Perdew, K. Burke, M. Ernzerhof, Phys. Rev. Lett. **1996**, 77, 3865.
[5] A. Jain, G. Hautier, C. J. Moore, S. Ping Ong, C. C. Fischer, T. Mueller, K. A. Persson, G. Ceder, Comput. Mater. Sci. **2011**, 50, 2295.

[6] L. Wang, T. Maxisch, G. Ceder, Phys. Rev. B - Condens. Matter Mater. Phys. **2006**, 73, 195107.
[7] W. W. Gärtner, Phys. Rev. **1959**, 116, 84.
[8] J. Li, L. M. Peter, J. Electroanal. Chem. **1985**, 193, 27.

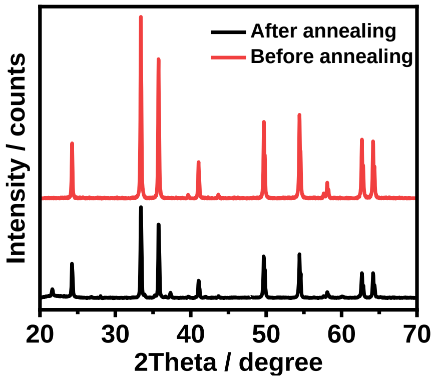


**Figure S1.** XRD patterns of GFO films before and after annealing at 600 °C.

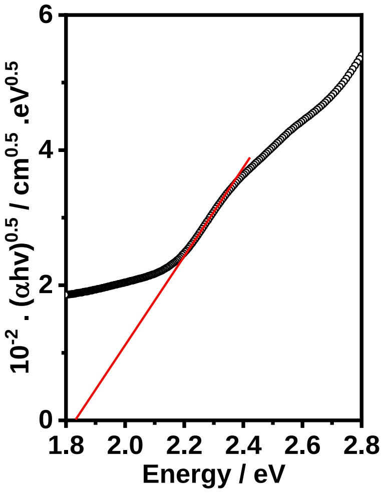


**Figure S2.** Indirect Tauc plot of GFO films.

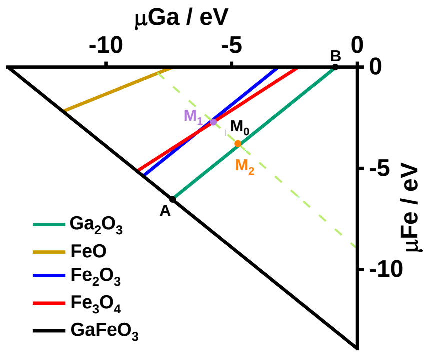


**Figure S3.** Thermodynamic stable chemical potential ranges for GFO with respect to competing phases.

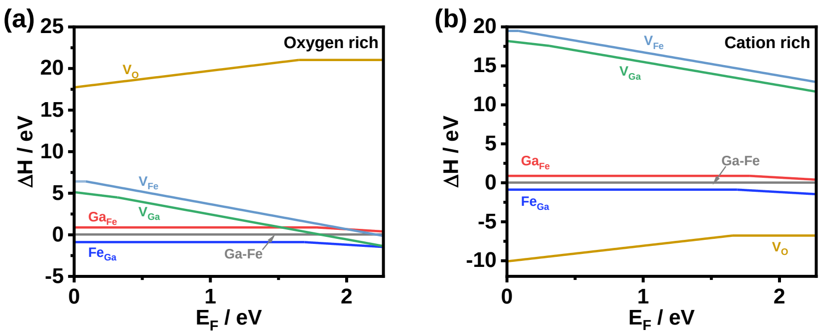


**Figure S4.** Defect formation energies of various intrinsic defects in GFO as a function of the Fermi level at possible extreme conditions: (a) oxygen rich / cation poor and (b) cation rich / oxygen poor.

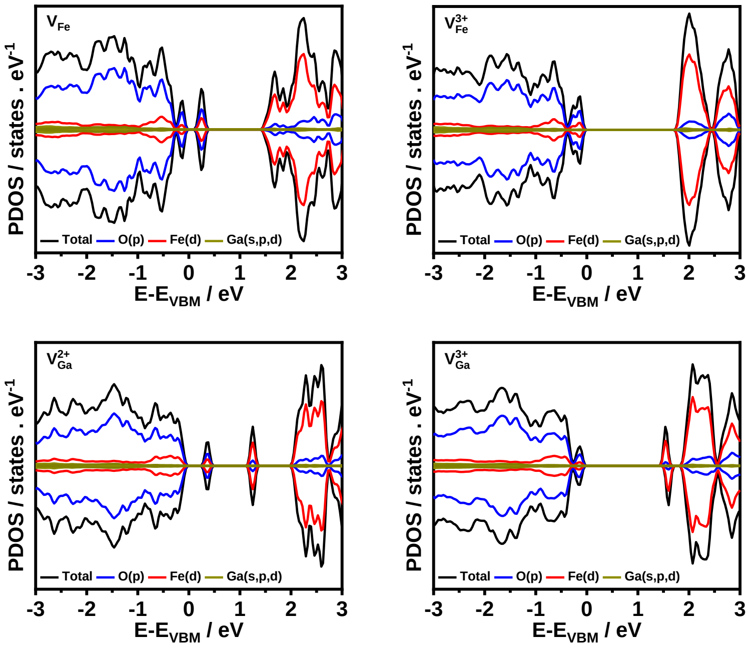


**Figure S5**. Projected density of states of rhombohedral of GFO with cation vacancy defects.

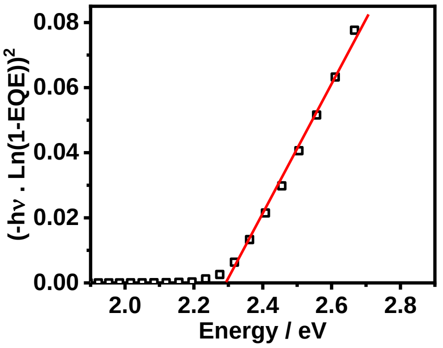


**Figure S6.** Tauc plot representation of the EQE spectrum (in the presence of $Na_2SO_3$) shown in Figure 3b.

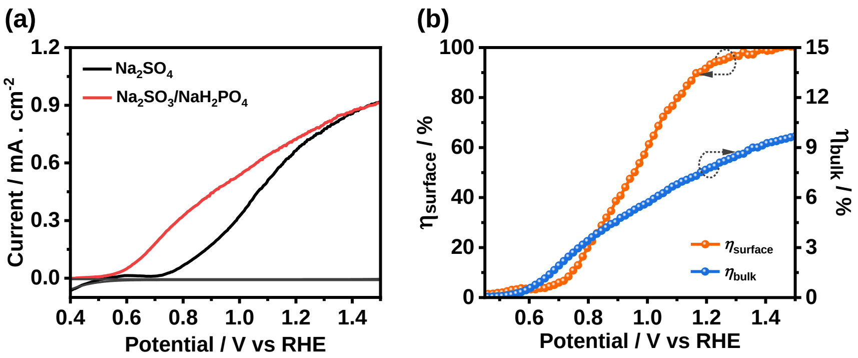


**Figure S7.** (a) photocurrent responses of GFO films in Ar-saturated 0.1 M $Na_2SO_4$ and 0.1M $Na_2SO_3/NaH_2PO_4$ solutions under continuous illumination of AM 1.5G (100 mW $cm^{-2}$) and (b) corresponding charge transfer and transport yields.

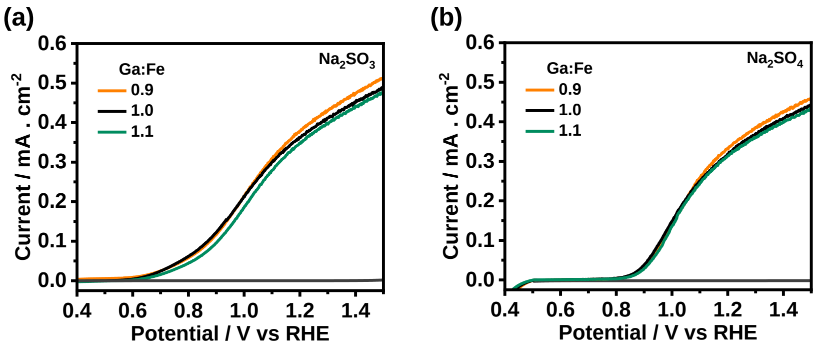


**Figure S8.** Photocurrent responses of GFO films with different cation ratio in Ar-saturated 0.1 M (a) $Na_2SO_4$ and $Na_2SO_3$ (b) electrolytes at pH 12 under continuous illumination of AM 1.5G (100 mW $cm^{-2}$).

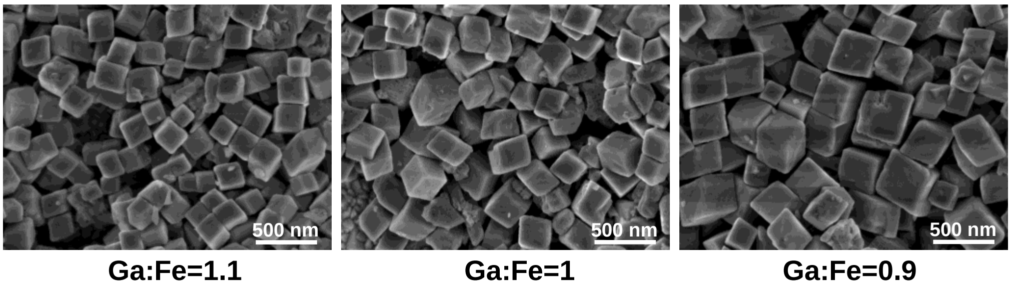


**Figure S9.** Top view SEM images of three GFO films prepared by spin-coating nanoparticle suspension onto FTO substates.

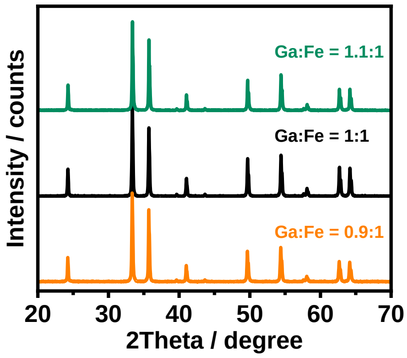


**Figure S10.** XRD patterns of GFO with different compositions.

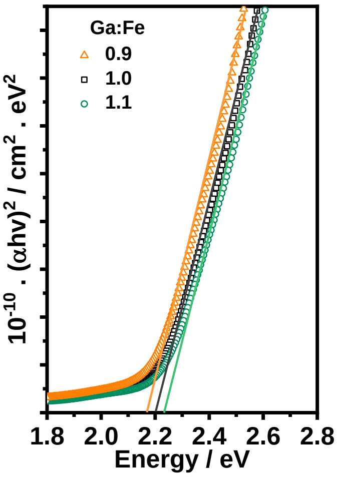


**Figure S11.** Tauc plots of nanoparticle-based GFO films with different cation ratio.

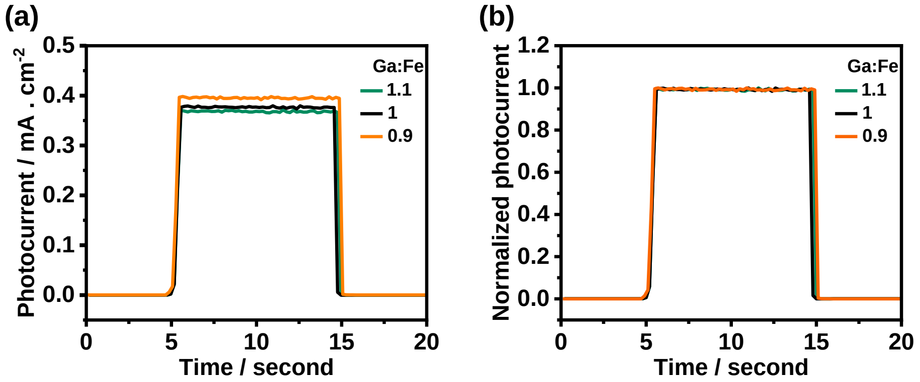


**Figure S12.** (a) transient photocurrent responses of various GFO films and (b) corresponding normalized photocurrent in Ar-saturated 0.1M $Na_2SO_3$ aqueous solutions (pH 12) under AM 1.5G illumination at 1.23 V vs RHE.

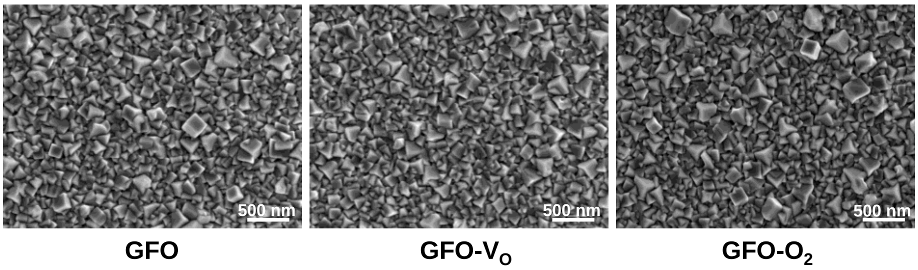


**Figure S13.** Top view SEM images of GFO films prior and after thermal reduction and oxidation.

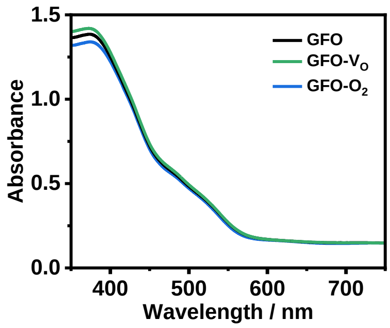


**Figure S14.** UV-vis absorption spectra of GFO, GFO-$V_O$ and GFO-$O_2$ thin films.

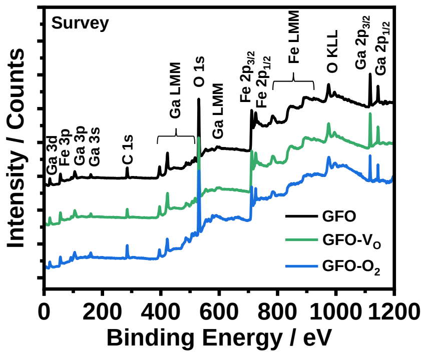


**Figure S15.** Survey XPS spectra of GFO, GFO-$V_O$ and GFO-$O_2$ thin films.

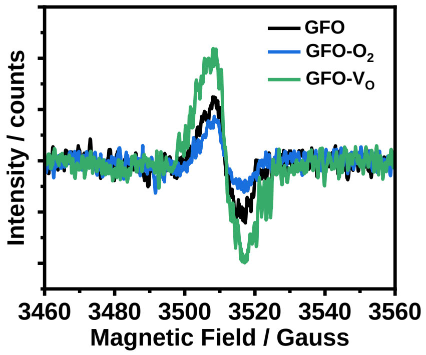


**Figure S16.** EPR spectra of GFO, GFO-$V_O$ and GFO-$O_2$ films at room temperature.

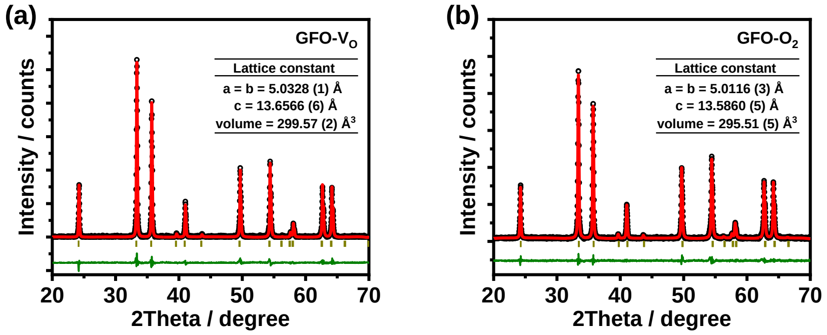


**Figure S17.** Rietveld refined XRD of GFO after (a) Ar and (b) $O_2$ treatment. The structure symmetry of GFO-$V_O$ and GFO-$O_2$ is identical with pristine GFO, and no other phases are observed. The inset shows the lattice parameters.

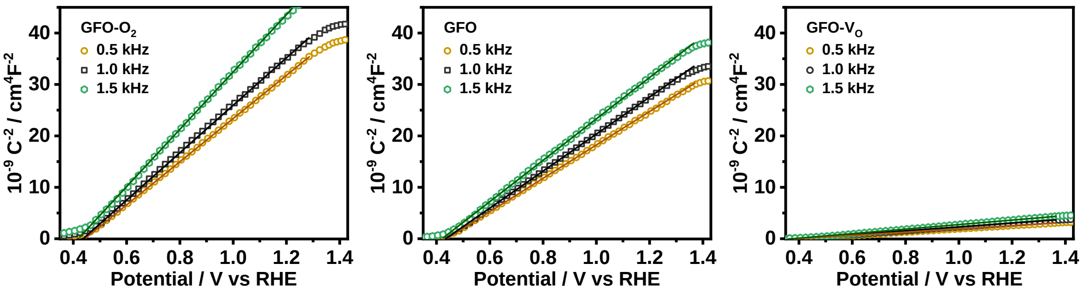


**Figure S18.** Mott-Schottky plots of three GFO films recorded at different frequencies. Other experimental conditions are the same as in Figure 4d.

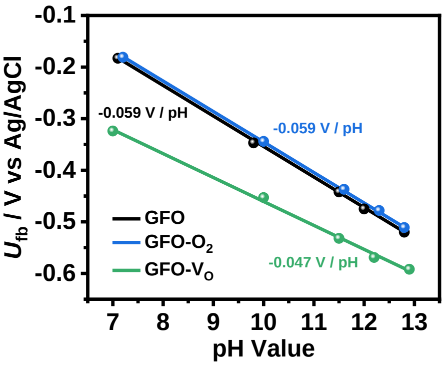


**Figure S19.** Solution pH dependence of flat band potential. These $U_{fb}$ values were obtained from Mott-Schottky equation. As discussed in the main text, the estimated $U_{fb}$ value of GFO and GFO-$O_2$ films is reliable. On the other hand, the determination of $U_{fb}$ for GFO-$V_O$ films using Mott-Schottky plots is affected by Fermi level pinning, introducing significant uncertainties. For example, the $U_{fb}$ value of GFO-$V_O$ films can fluctuate in the range of 0.35 – 0.52 V vs RHE at pH 12, depending on the selection of linear region range. Considering the linear region choice of other Ar-treated GFO films, 0.35 V vs RHE was used as the $U_{fb}$ of GFO-$V_O$ films. However, it is unambiguous that the $U_{fb}$-pH plot of GFO-$V_O$ films exhibits a slower change (smaller absolute value of slope) than that of GFO and GFO-$O_2$ films.

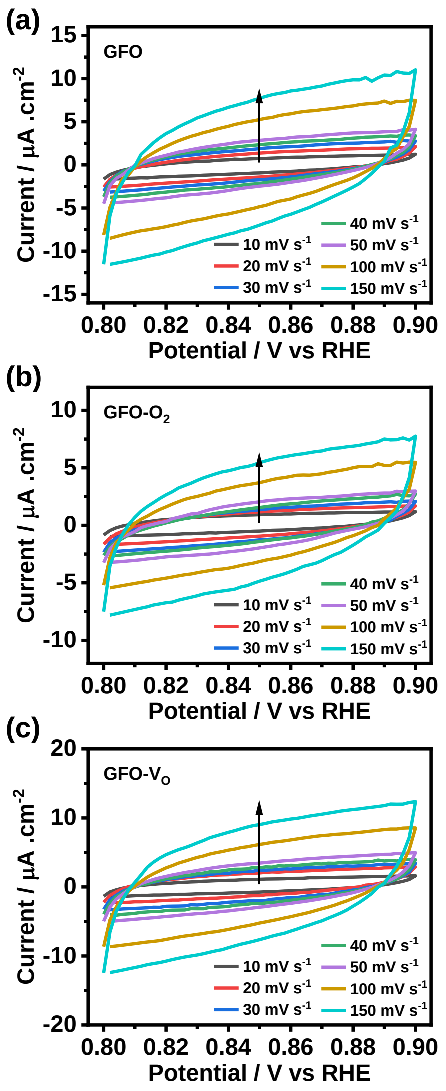


**Figure S20.** Cyclic voltammograms of (a) GFO, (b) GFO-$O_2$ and (c) GFO-$V_O$ films recorded at scan rates between 10 and 150 mV $s^{-1}$ in 0.1 M Ar-saturated $Na_2SO_4$ at pH 12.

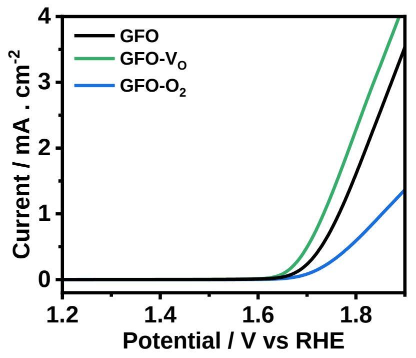


**Figure S21.** Electrocatalytic performance of three GFO films in 0.1 M Ar-saturated $Na_2SO_4$ at pH 12 in the dark.

**Table S1**. Chemical potential values of gallium, iron and oxygen at A, B and $M_{0-2}$ point.

| Point | Represented condition | μGa / eV | μFe / eV | μO / eV |
|---|---|---|---|---|
| A | cation-poor/oxygen-rich | -7.37 | -6.53 | 0.00 |
| B | cation-rich/oxygen-poor | -0.85 | 0.00 | -4.63 |
| $M_0$ | moderate cation and oxygen | -5.24 | -3.25 | -1.80 |
| $M_1$ | moderate cation and oxygen | -5.73 | -2.72 | -1.82 |
| $M_2$ | moderate cation and oxygen | -4.75 | -3.78 | -1.79 |

**Table S2.** Contrast between various Fe species content in pristine GFO, Ar-treated GFO and $O_2$-treated GFO obtained from the deconvolution of Fe $2p_{3/2}$ peaks (Figure 5b). It is noted that these values should be considered cautiously, as an approximate method was used for the deconvolution of Fe 2p signals.

| | GFO | GFO-$V_o$ | GFO-$O_2$ |
|---|---|---|---|
| $Fe^{2+}$ content / % | 23.2 | 25.8 | 21.7 |
| $Fe^{3+}$ content / % | 54.6 | 54.0 | 54.3 |
| $Fe^{4+}$ content / % | 22.2 | 20.2 | 24.0 |

**Table S3.** The electron density ($N_d$) of various GFO films calculated using Mott-Schottky equation.

| | GFO-$O_2$-45min | GFO-$O_2$-30min | GFO-$O_2$-15min | GFO | GFO-Ar-1h | GFO-Ar-2h | GFO-Ar-3h |
|---|---|---|---|---|---|---|---|
| $N_d$ / $cm^{-3}$ | $8.51\times10^{17}$ | $9.2\times10^{17}$ | $1.04\times10^{18}$ | $1.08\times10^{18}$ | $2.24\times10^{18}$ | $2.93\times10^{18}$ | $1.02\times10^{19}$ |

**Table S4.** The fitting parameters ($R_{ct}$) of electrochemical impedance spectra

| | GFO | GFO-$O_2$ | GFO-$V_O$ |
|---|---|---|---|
| $R_{ct}$ / Ω | 930.42 | 973.07 | 1327.41 |
